# Draft Genome Sequences of *Pseudomonas moraviensis* UCD-KL30, *Vibrio ostreicida* UCD-KL16, *Colwellia* sp. Strain UCD-KL20, *Shewanella* sp. Strain UCD-KL12, and *Shewanella* sp. Strain UCD-KL21, Isolated from Seagrass

**DOI:** 10.1128/genomeA.00023-17

**Published:** 2017-03-30

**Authors:** Karley M. Lujan, Jonathan A. Eisen, David A. Coil

**Affiliations:** a University of California Davis Genome Center, Davis, California, USA; b Department of Evolution and Ecology, University of California Davis, Davis, California, USA; c Department of Medical Microbiology and Immunology, University of California Davis, Davis, California, USA

## Abstract

Here, we present the draft genome sequences for five bacterial strains. These strains were all isolated from seagrass (*Zostera marina*) collected from Bodega Bay, CA, as a part of an undergraduate research project focused on seagrass-associated microbes.

## GENOME ANNOUNCEMENT

As part of the seagrass microbiome project (https://seagrassmicrobiome.org/), bacterial isolates were cultured from seagrass (*Zostera marina*) and surrounding sediment. In order to determine which isolates would undergo genome sequencing, we used the general protocol for identifying isolates used by Dunitz et al. (1). *Pseudomonas moraviensis* UCD-KL30 was isolated from seagrass leaf scrapings placed in phosphate-buffered saline (PBS), which was plated onto nitrogen-free agar (15 g/liter agar, 1 g/liter CaCO_3_, 1 g/liter K_2_HPO_4_, 0.2 g/liter MgSO_4_, 0.2 g/liter NaCl, 0.1 g/liter FeSO_4_, 5 g/liter Na_2_MoO_4_, 50 ml 1:50 [wt/vol] glucose) and left them at 25°C for 2 weeks. The remaining isolates were cultured on Difco marine broth agar plates. Isolate *Shewanella* sp. UCD-KL12 was selected from a PBS rinse of a scraped seagrass leaf, which was cultured at 4°C for 3 weeks. Isolates *Colwellia* sp. UCD-KL20 and *Shewanella* sp. UCD-KL21 were obtained from a single dilution of seagrass sediment that was cultured for a week at 25°C. *Vibrio ostreicida* was selected from a PBS rinse of a seagrass leaf cultured for 2 days at 25°C. Kept at their respective temperatures, Difco marine broth was used to create liquid overnight cultures for all five isolates.

Following the genomic DNA extraction, to complete the whole-genome sequencing, paired-end libraries were created using a Nextera XT library preparation kit (Illumina). This size-selected library (600 to 900 bp) was sequenced on a paired-end 300-bp run of an Illumina MiSeq. Quality trimming error correction and assembly were performed using the A5-miseq assembly pipeline (2, 3). Genome completeness was estimated using PhyloSift, which revealed that each assembly contained single copies of 37 conserved single-copy marker genes (2). For all genomes, annotation was completed using RAST (4). The results for each assembly and annotation can be found in Table 1.

**TABLE 1:** Genome assembly information

Strain identifier	Accession no.	No. of contigs	Genome size (bp)	*N*_50 _(bp)	G+C content (%)	Coverage (×)	No. of coding sequences	No. of RNAs
*Shewanella* sp. UCD-KL12	MPHJ00000000	62	5,697,218	342,963	43.2	78	5,004	147
*Shewanella* sp. UCD-KL21	MPHK00000000	85	4,604,458	204,923	41.9	76	4,005	131
*Colwellia* sp. UCD-KL20	MPHL00000000	69	4,535,601	259,628	35.6	81	3,890	81
*Vibrio ostreicida* UCD-KL16	MPHM00000000	61	4,501,752	253,705	45.6	75	4,275	130
*Pseudomonas moraviensis* UCD-KL30	MQUK00000000	25	6,106,149	626,618	59.8	39	5,406	69

Full-length 16S rRNA gene sequences were retrieved from the RAST annotation and then used in RDP (5) to create alignments for each isolate and their close relatives. In order to determine the taxonomy, RDP was also used to obtain 16S rRNA gene sequences for close relatives and used in the creation of the phylogenetic trees (5). All trees were inferred using FastTree (6), visualized in Dendroscope (7), and can be found on Figshare (https://doi.org/10.6084/m9.figshare.4508978.v2 and https://doi.org/10.6084/m9.figshare.4235549.v1).

For the *Colwellia* and *Shewanella* isolates, the alignments were used to infer a maximum likelihood 16S rRNA tree using data from close relatives. For all three strains, the species-level taxonomy was ambiguous, and we did not assign species names to these isolates. Similar analysis for UCD-KL16 resulted in a well-supported clade that contained multiple other *V. ostreicidia* strains that were not found anywhere else in the tree. For UCD-KL30, a 16S rRNA gene phylogenetic tree proved to be uninformative. Therefore, we created a concatenated 37-marker tree of this strain and other sequenced relatives. The resulting tree reveals an error in taxonomy, a strain of *Pseudomonas koreensis* within a clearly delineated clade of *P. moraviensis*. We confirmed this misidentification using an average nucleotide identity (ANI) comparison of these strains (8).

### Accession number(s).

These genome sequences are available under the accession numbers provided in Table 1.
